# Triboelectric touch sensor for position mapping during total hip arthroplasty

**DOI:** 10.1186/s13104-020-05238-4

**Published:** 2020-08-26

**Authors:** Jae Bum Jeong, Hyeok Kim, Jun-Il Yoo

**Affiliations:** 1 Department of Electrical Engineering, RIGET, Gyeongsang National University, Jinju, 52828 Korea; 2 Department of Electrical and Computer Engineering, University of Seoul, Seoul, South Korea; 3 Department of Orthopaedic Surgery, Gyeongsang National University Hospital, 90 Chilamdong, Jinju, Gyeongnamdo 660-702 Republic of Korea

**Keywords:** Triboelectric touch sensor, Position mapping, Total hip arthroplasty

## Abstract

**Objective:**

In this research, a triboelectric nanogenerator (TENG) was utilized to determine if a pressure-based sensor could detect bearing friction in a total hip arthroplasty (THA) and detect the contact of specific areas during ROM checks.

**Results:**

The pressure-based sensor shows capability to sense bearing friction. In more detail, the TENG embedded in four different sides of the trial exhibits up to 1 V from peak-to-peak. Moreover, these flexible touch sensors with TENG describes a peak signal in output voltage which should lead to extremely sensitive detection of bearing friction induced by the THA.

## Introduction

Total hip arthroplasty (THA) has become a common treatment for end-stage osteoarthritis of the hip [1]. However, despite improved implant designs and surgical techniques, bearing surface wear and the resultant wear-induced osteolysis have been major limitations to long-term prosthesis survival [1–3]. In an attempt to avoid the problems caused by wear debris, hard bearing surfaces, such as ceramic-on-ceramic (CoC) have been developed.

In the decades since the 1970s, CoC bearings have made many advances. COC bearings have improved significantly in terms of wear reduction. However, concerns such as ceramic fracture, have not been resolved yet [4]. One of the biggest reasons for ceramic fracture is mal-seating of the ceramic liner [5, 6]. Yoshitoshi et al. reported that 20% of the liners were observed to be mal-seated in imaging studies [5]. The clinical outcome of liner mal-seating, however, was not determined because there was no long-term follow-up. Nevertheless, negative outcomes, such as osteolysis, may occur.

In addition, an intraoperative range of motion (ROM) check in the surgical field can predict postoperative impingement. And soft tissue tension and balance are measured by the Shuck test. All of the various postoperative problems depend on the experience and judgement of the surgeon in the operative field. Recently, Mecdessay et al. reported that the method for determining soft tissue balance used in total knee replacement arthroplasty (TKRA) was very inaccurate [7]. Moreover, they also noted that the use of pressure-based sensors increased the accuracy of knee balance determinations.

Inertial Measurement Units (IMU) based THA surgery has been reported in the experimental setting. However, there are few reports about sensor-based total hip implants in the real-life clinical field. This is because it is difficult to measure pressure and soft tissue balancing in spherical ball and socket joints.

The triboelectric nanogenerator (TENG) was recently been developed as an effective tool for converting mechanical energy generator by an organic/polymer nanogenerator (NG) into electricity. The TENG has attracted considerable interest in the field of conventional piezoelectric devices and has been applied to various applications, such as wearable devices, wireless, stretchable devices, sensors, and flexible electronics [8–13]. The TENG can also play roles as pressure and touch sensors by sensitive reactions based, not only on the large capacity for voltage generation but also the amount of electricity generated due to friction or pressure [9]. Pressure sensors can be used in a wide variety of applications, such as smart medical devices, real-time health status analysis, and operation modules for virtual reality control [12]. Moreover, most sensors require an external power supply, however, the TENG aims for wearable sensing devices because it can generate electricity and drive the sensor without any external power sources [14–16].

In this research, a TENG was utilized to determine if a pressure-based sensor could detect bearing friction in a THA and detect the contact of specific areas during ROM checks.

## Main text

### Methods

To fabricate the flexible switch sensor, polydimethylsiloxane (PDMS) was mixed well with base and hardener at a ratio of 10:1. Bubbles are formed in the PDMS during mixing, so treatment with a vacuum desiccator was performed to remove all bubbles from the PDMS. When all the bubbles were removed, the PDMS was poured onto a 4-inch Si wafer and spin-coated at 300 rpm for 30 s. Then, the PDMS was cured at 65° C for 2 h in an oven. The PDMS film was then removed from the Si wafer. Copper tape, which is made of metal, was attached to the produced PDMS film to make an electrode and connection was made with a wire.

**Fig. 1:**
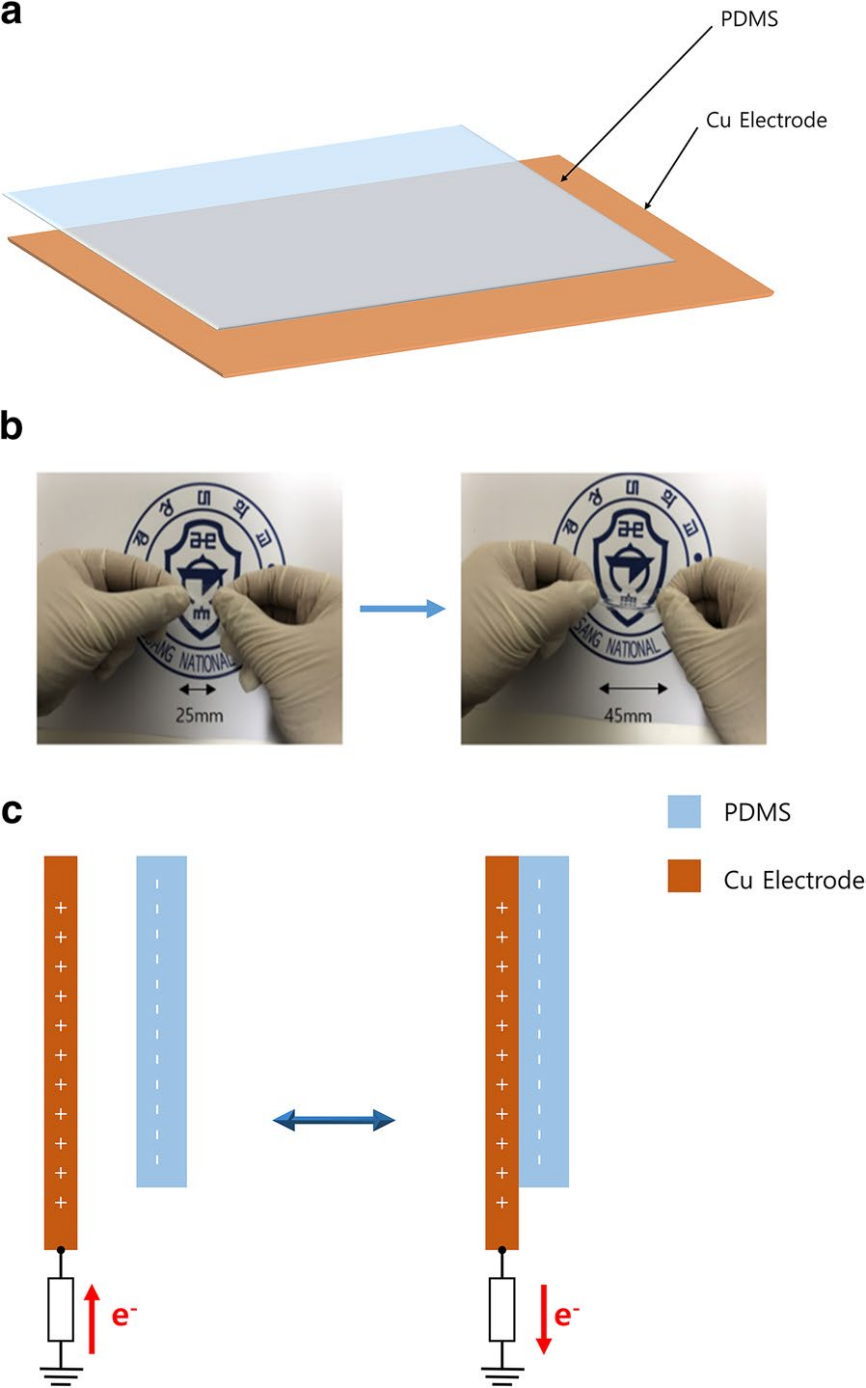
TENG sensor. **a** device structure, **b** PDMS, **c** operational mechanism of triboelectric switch sensor

The device structure of the TENG sensor described above is schematically shown in Fig. 1a. In this configuration, the TENG worked through frictional triboelectricity by touching/untouching induced from deformation (Fig. 1b, c).

**Fig. 2:**
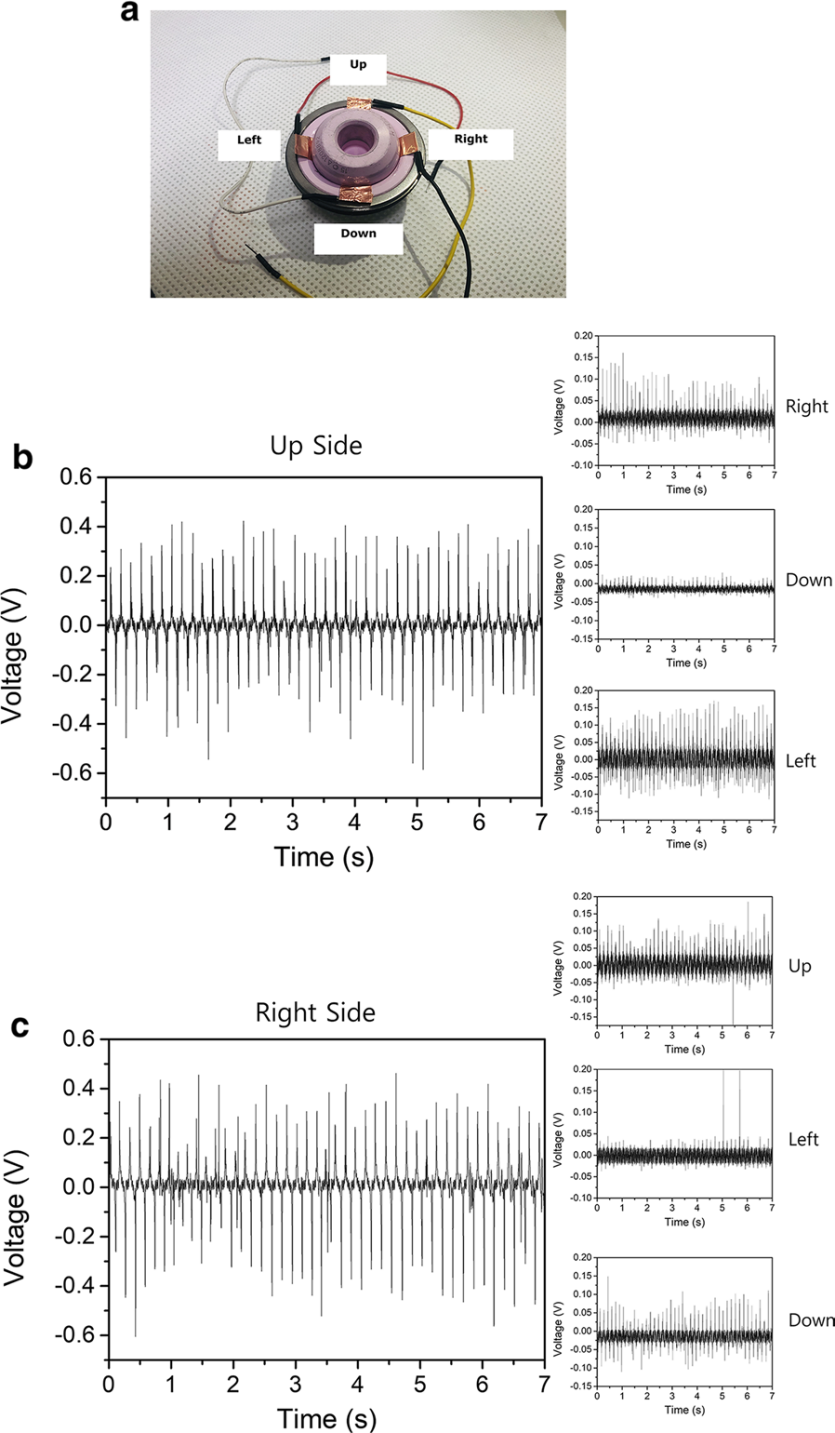

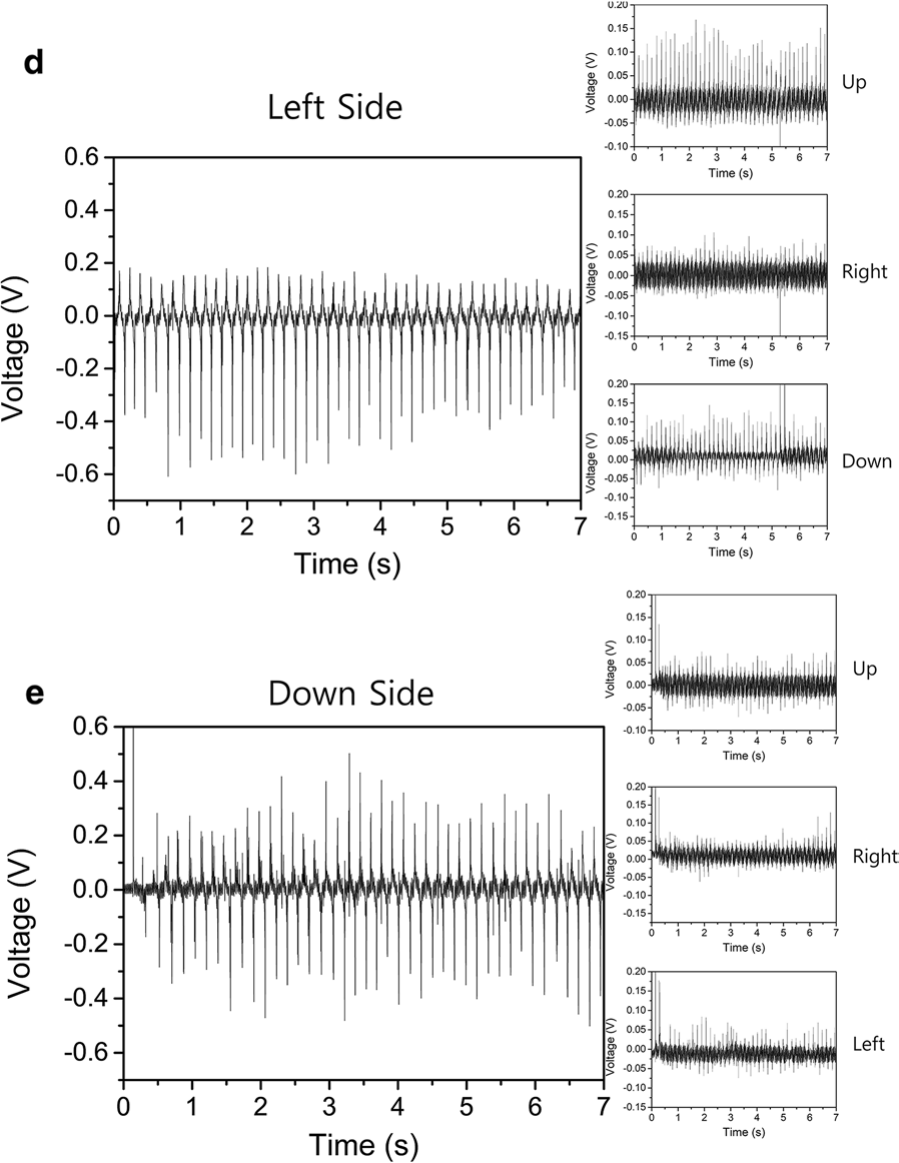
**a** Photograph of flexible touch sensors-embedded trial. **b** Output voltage of measured signal on upside; Output voltage of measured signal on **c** right, **d** left, **e** down sides

To demonstrate the output characteristics of a TENG, a TENG-embedded trial was designed as depicted in Fig. 2a. Figure 2b–e show the results obtained by measuring the output voltage with respect to time evolution by the application of touch between the TENG and the trial in all four directions. While the trial moved, a deformation appeared in the TENG and the bottom surface of the PDMS attached to the copper tape produced and lost triboelectric surface charges repeatedly. As a result, the output voltage emerged at the external circuit of the TENG. We measured these output voltages with an oscilloscope (KEYSIGHT DSOX2014A).

### Results and discussion

The pressure-based sensor was able to sense bearing friction. In more detail, the TENG embedded in four different sides of the trial showed up to 1 V from peak-to-peak which was large enough to differentiate the detected signal from a noise level less than 0.1 V. Moreover, these flexible touch sensors with TENG exhibited a peak signal in output voltage which should lead to extremely sensitive detection of bearing friction induced by the THA. In general, pressure sensors, which are based on piezo-resistive active material or semiconductor materials fabricated by micro-electromechanical systems (MEM) technology, describe a continuous signal from external pressure. This may lead to insensitive detection between diverse motions on the THA. In contrast, the TENG sensor system enabled comparably high sensitivity due to its capability to produce an output voltage with peaks.

The pressure-based sensor was able to detect contact in certain areas while the COC bearing was in ROM. To investigate how the other sensors were affected when we applied pressure to one of four sensors in the THA, we measured the noise signals of the other three sensors, as depicted in the insets of Fig. 2b–e. The insets show the noise signals in the output voltage from the three other sensors when pressure was applied to the other sensor. The noise level rose as high as 0.2 V, which was negligible compared to the measured voltage output of the sensor in the target position (up-side) in Fig. 2b. The same phenomenon observed in Fig. 2c–e was seen for the right, left, and down sides respectively. These results show that sensing orthogonality was completely guaranteed by this device.

The principal findings were that the TENG pressure-based sensor was able to detect bearing friction in the THA and that it was able to detect the contact area of ​​the bearing surface during ROM.

Soft tissue balancing is a very important test to prevent hip dislocation and to decrease postoperative pain in THA [17]. Until now, it has been evaluated subjectively by the operator using such tools as the Shuck test [17]. However, an objective evaluation of the pressure sensor used in this study will increase the success rate of the surgery.

In the case of total knee arthroplasty, a soft tissue balance check using a pressure-based sensor was conducted in the clinical field and was shown to be highly reproducible compared to the hand check [7].

If such sensor base data accumulates, it will be possible to explain the post-operative dislocation or complications that are unknown. In addition, better postoperative results can be expected by using pressure data, as well as imaging data, to determine the length of legs during surgery. When the sensor is inserted into the body, real-time wear monitoring becomes possible and the data can be used to analyze the cause of the revision timing and the pain of the patient. Furthermore, it is expected that research on sensors will be carried out at various implant development stages.

In the range of motion evaluation during surgery, it is possible to predict the risk of impingement and dislocation by observing an increase in pressure at a specific area of the bearing surface. That is, it will be possible to immediately change the implant location within the surgical field. In addition, pressure sensing, and specific area mapping techniques will be used to determine the position of the ceramic liner and reduce the risk of mal-seating. In the future, the TENG sensor in THA will help soft tissue balancing and intraoperative ROM measurement. In particular, as the material science develops due to the characteristic of the self-powered TENG sensor, direct implantation into the articulation will be possible. In addition, the future integration of sensors that quantify the patient’s soft tissue tension, and hip stability through a full range of motion, enables the robot to make incremental implant and bone readjustments to allow true customization of a patient’s total hip soft tissue balance and alignment.

The conclusion of this study is that the TENG pressure-based sensor was able to detect friction in the THA bearing and detect the contact area of the bearing surface in the ROM.

Further research will be carried out to develop biocompatible sensors and to enable precise pressure-sensing.

## Limitations

There were several limitations to this study. First, finer resolution was not able to scale the pressure. Second, we could not fine-tune the sensor mapping while guaranteeing orthogonality. Third, biotoxicity and biocompatibility studies should be performed to develop biodegradable formulations. And last, the accuracy and cost-effectiveness should be compared with existing navigation equipment.

## Data Availability

The dataset supporting this article is available upon request; please contact the corresponding author.

## Abbreviations

TENG: triboelectric nanogenerator
THA: total hip arthroplasty
PDMS: polydimethylsiloxane
ROM: range of motion
CoC: ceramic-on-ceramic
TKRA: total knee replacement arthroplasty
IMU: inertial Measurement Units
